# The Diagnosis of Early Consumption by the X-Rays

**Published:** 1907-05-11

**Authors:** C. Willett Cunnington


### The General Practitioners' Column.

### THE DIAGNOSIS OF EARLY CONSUMPTION BY THE X-RAYS.

By C. WILLETT CUNNINGTON, B.A., M.B., B.C.(Camb.).

At present it would seem that the *x*-rays are regarded by general practitioners as a weapon essentially belonging to the specialist. This should not be so. Practitioners are apt to suppose that the chief use of *x*-rays is to examine bones and their lesions. The fact that by *x*-rays almost all diseased conditions within the thorax can readily be diagnosed is not sufficiently appreciated. Early consumption is usually diagnosed by certain symptoms coinciding with certain signs revealed by the stethoscope. Yet we must try to diagnose the disease at an earlier stage, for physical signs imply that it has gained a firm hold. With *x*-rays we can diagnose consumption before the stethoscope tells anything.

It should be recognised that all "doubtful chests" should be examined by the *x*-rays before they can be passed as healthy. The methods of *x*-ray examination are easy, but of course experience in working is needful. Yet this is equally true of the stethoscope.

### Methods of Examination.

We examine a chest by two methods in *x*-ray work. With the fluorescent screen the thorax can be examined in its movements, while with a photographic plate we can get a picture of "still life." Both should be employed in doubtful cases. With the screen we pay attention chiefly to three points:—

(1) *The Movement of the Diaphragm.*—If one lung is diseased, the corresponding half of the diaphragm moves through a smaller excursion than the other. This will occur even though no area of disease is seen; in other words, it occurs in extremely early cases.

(2) The degree of transillumination of the apices. A diseased apex will "light up" less well on deep inspiration than on the healthy side.

(3) We look for an area of shadow present both in inspiration or expiration. If such is found the disease has made some progress; this is not such an early sign, then, as the two first.

By means of the photograph we obtain impressions of very small areas of disease, too small to show on screen examination. Spots of disease usually first appear not at the extreme apex, but about the level of the fourth interspace. These are in cases presenting no physical signs to the stethoscope. When a case is under treatment it is helpful if a series of plates is taken at intervals of a few months; they serve as permanent records of the progress of the disease.

As an example, an anæmic patient, in whom there were no physical signs, showed impaired movement of the left diaphragm, and a doubtful spot in the fourth left inspace on the plate, in August. In the January following the doubtful spot was more obvious, and in February impaired resonance began to develop. Thus the *x*-rays revealed signs of disease some six months before the stethoscope. The natural cure of phthisis is by fibrosis of the diseased area, and this change is very apparent when a series of plates is examined. The spot on the plate becomes denser and denser; this is extremely useful in helping the observer to form a prognosis. A very important point in pulmonary tuberculosis is the fact that in many cases the mediastinal glands are early affected. The *x*-rays show that many cases of consumption begin by glandular enlargement, particularly at the root of the lungs. The disease can be seen to spread, as time goes on, into the upper lobe along its base, and only after some time reaching the extreme apex. It is only when this point is reached that stethoscopic signs occur, simply because it is the extreme apex of the lung which is nearest to the surface.

It is clear, then, that in many cases the disease begins, not at the apex, as is generally taught, but in the lower part of the upper lobe. Another important point is revealed by the rays—namely, that in all cases where there are physical signs at one apex the disease can be seen by the rays to exist already in both lungs.

This is only natural, if we remember that tuberculosis spreads by the lymphatics, starting, as I believe, almost always in the mediastinal glands.

I believe that "inhalation" consumption seldom occurs, and that the tubercle bacilli travel by the lymphatics. To say that "only one lung is affected" means "signs of the disease in the other lung are not yet audible"; the disease is there, nevertheless.

### The Mediastinal Glands.

A good deal of attention is paid, nowadays, to the condition of the mediastinal glands, and for the reasons above stated.

These glands are best examined by the screen. Each mediastinum can be readily examined in turn by altering the position of the patient. In the "right oblique" position we get an excellent view of the posterior mediastinum, which appears as a triangular light space, bounded by the heart in front and above, the aorta and spine behind, and the diaphragm below. This area in health is clear, and becomes markedly luminous on deep inspiration. When the retro-cardiac glands are enlarged the space remains dark. This phenomenon occurs very often in sickly anæmic children, and we cannot be surprised when, only too often, such children ultimately fall victims to consumption. They are described as having "a tendency to consumption." The *x*-rays tell us that in reality they already carry the germs of the disease within them. It will be seen, then, from these remarks that we have a most valuable instrument in helping us to solve a most difficult problem. We must no longer leave a patient labelled "Query early consumption?" We must look for the answer by the light of the *x*-rays, remembering that "coming events cast their shadows before."

